# Identification of small molecule inhibitors of the Aurora-A/TPX2 complex

**DOI:** 10.18632/oncotarget.16738

**Published:** 2017-03-31

**Authors:** Italia Anna Asteriti, Frederick Daidone, Gianni Colotti, Serena Rinaldo, Patrizia Lavia, Giulia Guarguaglini, Alessandro Paiardini

**Affiliations:** ^1^ Institute of Molecular Biology and Pathology, CNR National Research Council, Sapienza University of Rome, 00185, Rome, Italy; ^2^ Department of Biochemical Sciences, Sapienza University of Rome, 00185, Rome, Italy; ^3^ Department of Biology and Biotechnology, Sapienza University of Rome, 00185, Rome, Italy

**Keywords:** Aurora-A kinase, TPX2, protein-protein interactions, small molecule inhibitors, anti-cancer therapy

## Abstract

Aurora kinases are a family of cell division regulators that govern the correct assembly of a bipolar mitotic spindle and the fidelity of chromosome segregation. Their overexpression is associated with genomic instability and aneuploidy, and is frequently observed in cancer. Accordingly, competitive inhibitors targeting Aurora kinase activity at the ATP-binding site are being investigated for therapeutic purposes. Despite promising pre-clinical data, these molecules display moderate effects in clinical trials and incomplete selectivity, either against distinct family members, or other kinases. As an alternative approach, protein-protein interaction inhibitors targeting mitotic kinases and their activators can be exploited to achieve increased specificity of action. In this study, a virtual screening of small molecules led to the identification of 25 potential inhibitors of the interaction between Aurora-A and its activator TPX2. *In vitro* experiments confirmed that 4 hits bind Aurora-A in the low micromolar range and compete for TPX2 binding. Immunofluorescence assays showed that 2 compounds also yield lowered Aurora-A activity and spindle pole defects in cultured osteosarcoma cells. The identified protein-protein interaction inhibitors of the Aurora-A/TPX2 complex might represent lead compounds for further development towards pioneering anti-cancer drugs and provide the proof-of-concept for a new exploitable strategy to target mitotic kinases.

## INTRODUCTION

Targeting mitosis is a well known strategy to kill cancer cells: besides anti-microtubule (MT) drugs commonly used in chemotherapy, inhibitors of specific mitotic regulators are being developed to overcome the issues of toxicity and/or acquired resistance displayed by anti-MT molecules [1]. Aurora kinases play key roles in control of spindle function and mitotic progression [2]. Three members of the family (Aurora-A, -B and -C) have been described in mammals, with Aurora-C function restricted to meiotic division. The Aurora-A kinase mainly localizes at centrosomes and spindle poles and regulates nucleation, organization and function of spindle MTs. Aurora-B instead localizes at chromosomes, centromeres and central spindle, playing a key role in the correction of erroneous kinetochore-MT attachments and in the cytokinesis process [2–4]. Aurora kinases are among the most attractive targets for novel anti-cancer approaches, based on their frequent overexpression in tumors and on the anti-proliferative effects associated with their inactivation [3, 4].

All Aurora inhibitors that have entered clinical trials compete with ATP at the active site pocket (type I inhibitors), which is highly conserved, both structurally and evolutionarily, among human kinases [5, 6]. Limited selectivity is therefore a major hurdle, representing a bottleneck in the clinic and challenging type I inhibitors success. Single cell microscopy analyses recently showed that the most specific Aurora-A inhibitor in clinical trials, MLN8237 (Alisertib), has in fact still incomplete specificity towards the related Aurora-B [7, 8]. This can yield undesired effects, including lack of sustained mitotic arrest and exit from mitosis with unbalanced chromosome segregation, a condition that may contribute to pro-tumorigenic effects [7]. These issues may account, at least in part, for the observed gap between the promising pre-clinical results obtained with this inhibitor and the modest outcomes from clinical trials [3].

Kinases often bind specific protein partners at allosteric sites to gain full activity: novel classes of protein-protein interaction (PPI) inhibitors targeting pockets remote from the ATP-binding site are therefore rapidly moving to the forefront of kinase inhibitors research [9]. Such allosteric modulators bind to sites that are much less conserved across the kinome, and that are often only accessible upon conformational changes. Besides selectivity, allosteric PPI inhibitors of kinases offer additional advantages, e.g., insensitivity to the high intracellular ATP concentration, and expansion of the molecular repertoire of potentially effective scaffolds [10]. The best characterized Aurora-A activator is the MT-binding protein TPX2 (Targeting Protein for Xklp2). The interaction with TPX2 regulates Aurora-A activity at multiple levels: first, TPX2 is required for Aurora-A association to spindle MTs [11, 12]. Second, TPX2 binding increases Aurora-A kinase activity by protecting the key p-Thr288 in the kinase activation loop from the Protein Phosphatase 1 (PP1) and stabilizing the active conformation of the catalytic domain [13]. Residues 1-43 of human TPX2 are necessary and sufficient for Aurora-A binding, activation, and protection from dephosphorylation [13]. Actually, this is achieved via two distinct peptide stretches in TPX2 that bind Aurora-A at two different sites: the “upstream” peptide (residues 7-21 of TPX2) binds at the N-terminal lobe of the Aurora-A kinase domain, while the “downstream” α-helix (residues 30-43) binds Aurora-A between the N- and C-terminal lobes. The two Aurora-A binding peptides of TPX2 are connected by a flexible linker (disordered in the crystal structure) that is variable in length and sequence across species [13]. Finally, TPX2 also contributes to Aurora-A protein stability, by protecting it from proteasome-dependent degradation in early mitosis [14]. Thus, targeting the interaction between Aurora-A and TPX2 with small molecules may provide several advantages over ATP-competitors: it is expected not only to achieve kinase inhibition but also to lower Aurora-A protein levels. Most importantly, TPX2 specifically binds Aurora-A and not Aurora-B due to a single amino acid difference within the catalytic domain [15, 16], suggesting that such PPI inhibitors would at least partially overcome the specificity issues of ATP-competitors. Finally, TPX2 is frequently co-overexpressed with Aurora-A in cancer [17], suggesting that the complex may constitute a novel therapeutic target.

Here, starting from the available structural information on the interaction interface between the catalytic domain of Aurora-A and the first 43 amino acids of TPX2 [13], we have employed a virtual screening approach to search for inhibitors of the Aurora-A/TPX2 interaction. We describe the identification of two drug-like small molecules, showing the ability to interfere with the formation of the complex *in vitro* and to perturb Aurora-A activity and spindle structure in cultured osteosarcoma cells. In the search for a new generation of more specific and effective inhibitors of Aurora-A activity, these compounds represent promising scaffolds for future hit-to-lead optimization studies.

## RESULTS

### Analysis of the Aurora-A/TPX2 interaction interface and hot spots identification

The crystal structure of the human Aurora-A kinase domain (residues 122-403) bound to the 1-43 TPX2 fragment is available [13]. In order to develop the rational design of small molecule inhibitors of the Aurora-A/TPX2 interaction, we first in-depth investigated the key structural determinants of affinity and specificity at protein-protein interface (hot spots of interaction). To this end, two independent complementary approaches, i.e., evolutionary and thermodynamic analyses, were carried out using Consurf [18], CAMPO [19] and computational Alanine Scanning Mutagenesis (ASM) [20]. The evolutionary conservation values obtained from CAMPO and Consurf were normalized within a conservation score scale (0, highly variable; 9, invariant). Computational ASM predicted the change in binding free energy of Gibbs (ΔΔG) for the replacement of an amino acid side chain with Alanine. Positive and negative ΔΔG values are indicative of a destabilizing or stabilizing effect, respectively, upon mutation.

**Figure 1:**
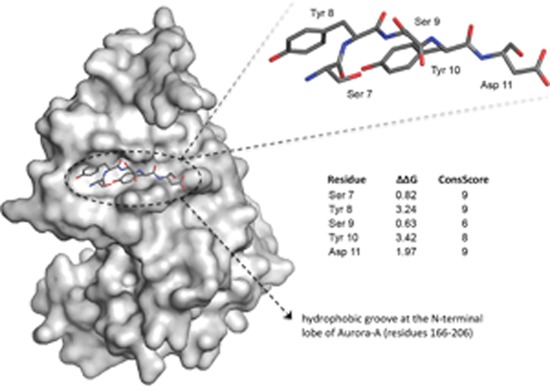
Analysis of the Aurora-A/TPX2 interaction interface and hot spots identification Residues 7-11 of human TPX2 (sticks) bind at a shallow hydrophobic groove at the N-terminal lobe of Aurora-A (grey surface). Evolutionary conservation (ConsScore) and ΔΔG upon computational Alanine mutagenesis are reported. Among these residues, Tyr 8 (ΔΔG = 3.24 Kj/Mol) and Tyr 10 (ΔΔG = 3.42 Kj/Mol) were predicted as key residues for the thermodynamic stabilization of the complex.

The results obtained from evolutionary and thermodynamic analyses were mapped onto the crystal structure of the TPX2 7-21 and 30-43 peptides to identify conserved clusters of residues that are primarily involved in the stabilization of the complex with Aurora-A. Residues 7-11 of the “upstream” stretch of TPX2, which bind at a shallow hydrophobic groove at the N-terminal lobe of the kinase, were assigned top scores for evolutionary conservation. Among the top evolutionarily scoring residues, Tyr 8 (ΔΔG = 3.24 Kj/Mol) and Tyr 10 (ΔΔG = 3.42 Kj/Mol) were considered key residues for the interaction, as defined by Moreira et al. (conserved residues with binding free energy differences between 2.0 and 4.0 kcal/mol) [20]. Residues 7-11 of TPX2 are thus evolutionarily conserved, as well as predicted to be particularly important for the thermodynamic stabilization of the complex (Figure 1). These data, therefore, stress the importance of peptide 7-11 of TPX2 (TPX2-7-11) as hot spot of interaction with Aurora-A.

### Pharmacophore hypothesis and virtual screening for potential inhibitors of the Aurora-A/TPX2 interaction

**Figure 2:**
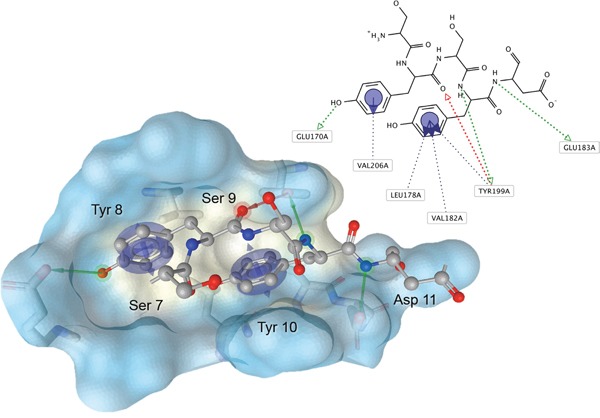
Pharmacophore hypothesis of the Aurora-A/TPX2-7-11 interaction Residues 7-11 of human TPX2 (balls-and-sticks) were identified as a hot spot of interaction and used to derive a PH (arrows for projections and centroids) at a shallow hydrophobic groove at the N-terminal lobe of Aurora-A (surface, colored by hydrophobicity). Green arrow, hydrogen-donor feature; red arrow, hydrogen-acceptor feature; blue arrow/centroid, hydrophobic/aromatic interaction. Exclusion volumes are not shown. This figure was rendered with LigandScout (Inte:Ligand).

The set of structural features of TPX2-7-11 that are directly related to Aurora-A recognition have been exploited to derive a protein-based pharmacophore hypothesis (PH; Figure 2). A pharmacophore query was used to build a 12-points PH, along with exclusion volumes, involving six chemical moieties: (1) an aromatic centroid located at the geometric center of the aromatic ring of Tyr 8, and its normal projection, which points at Val 206; (2) a hydrogen bond donor feature located on the hydroxyl moiety of Tyr 8, and its projection, which points at the side chain of Glu 170; (3) an aromatic centroid located at the geometric center of the aromatic ring of Tyr 10, and its normal projection, which points at a groove formed by Leu 178, Val 182 and Tyr 199; (4) a hydrogen bond donor feature located on the main-chain N atom of Tyr 10, and its projection, which points at the side chain of Tyr 199; (5) a hydrogen bond donor feature on the main-chain of Asp 11, and its projection, which points at the side-chain of Glu 183; (6) a hydrogen bond acceptor feature located on the oxygen of the carbonyl group of Tyr 8, and its projection, which points at the side chain of Tyr 199. Finally, in order to take into account the shape of the binding site of Aurora-A, a steric constraint (excluded volumes) was derived from the Aurora-A/TPX2-7-11 complex, and included in the final PH. The latter was used to screen the drug-like (~5×10^6^ compounds) and lead-like (~3×10^6^ compounds) subsets of the ZINC database [21]. All the features of Tyr 8 and Tyr 10 were set as essential; compounds not satisfying at least three features of the PH were discarded. At the end of this process ~6×10^5^ compounds (~5×10^5^ drug-like and ~1×10^5^ lead-like) were kept. These virtual hits underwent a second docking-based filtering by means of the Dovis docking tool [22]. All potential hits were docked into the TPX2-7-11 binding site of Aurora-A, and the normal distribution of their scores (reported as predicted pKi values) was derived. The distributions of scores for lead-like and drug-like molecules showed a mean pKi of 5.10±0.21 and 5.89±0.93, respectively. 1032 drug-like and 2413 lead-like molecules with significant scores (≥2 standard deviations from mean) were selected and resubmitted to a further docking step by means of two additional docking tools, AutoDock Vina [23] and MolDock SE [24]. Of these, 43 lead-like and 130 drug-like molecules showed comparable poses when docked with Dovis, Vina and MolDock (RMSD value ≤2 Å), which fulfilled also the restraints imposed by the final PH. Similar compounds were clustered, and, based on the predicted pKi and the commercial availability, the most potent representatives of each group were chosen. This protocol yielded a total of 25 compounds that were subjected to *in vitro* testing (Table 1).

**Table 1:** List of compounds tested in this study

Code	ZINC Code	MW	LogS	Structure	Kd
**C01**	03194268	315	-6.1	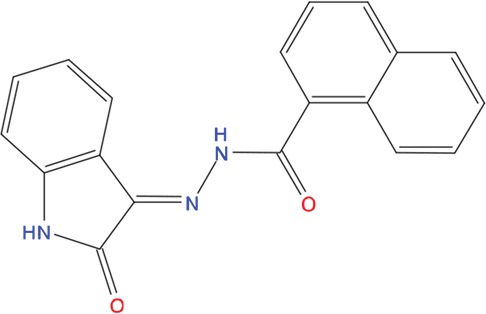	>100 μM
**C02**	05093481	316	-4.9	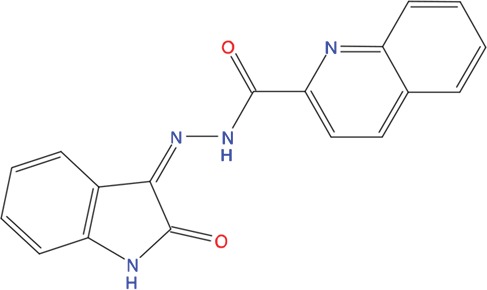	42±9 μM
**C03**	19598330	321	-4.4	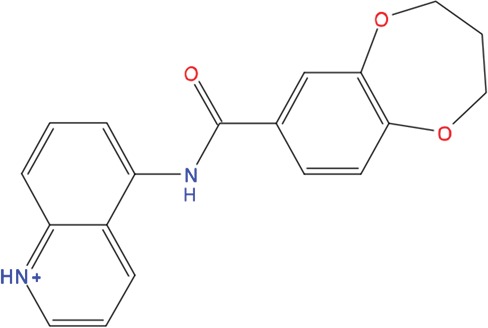	>100 μM
**C04**	05731657	323	-4.5	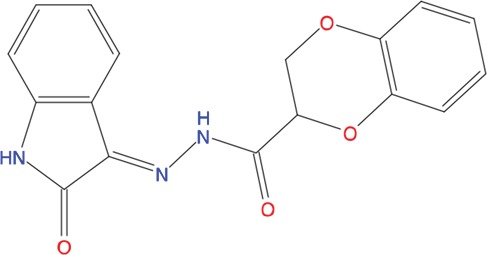	100 μM
**C05**	16698672	327	-4.4	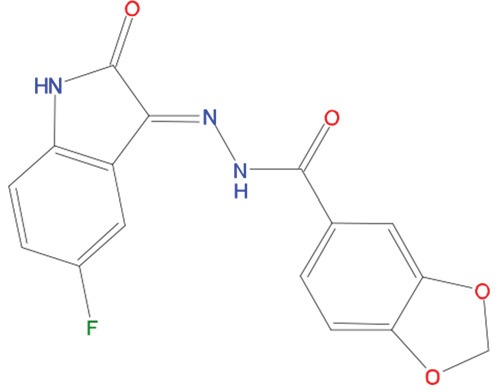	>100 μM
**C06**	12560933	330	-3.7	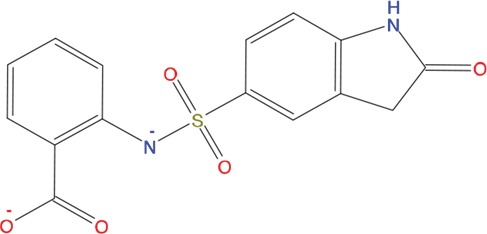	>100 μM
**C07**	06556932	337	-4.9	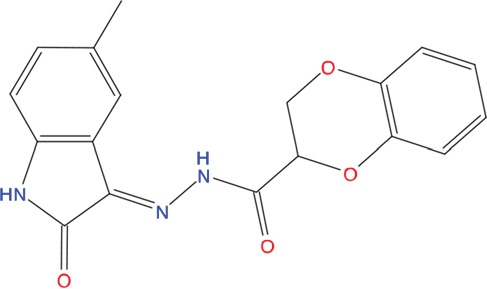	>100 μM
**C08**	09575811	338	-4.3	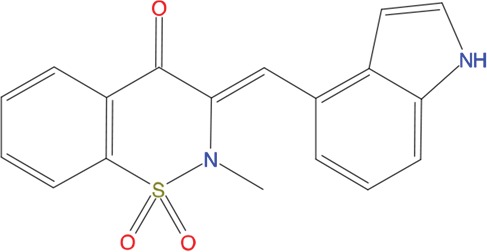	80±30 μM
**C09**	00521297	344	-5.6	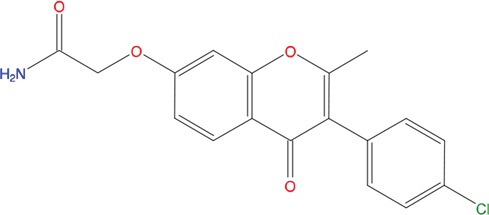	80±15 μM
**C10**	14240291	346	-5.5	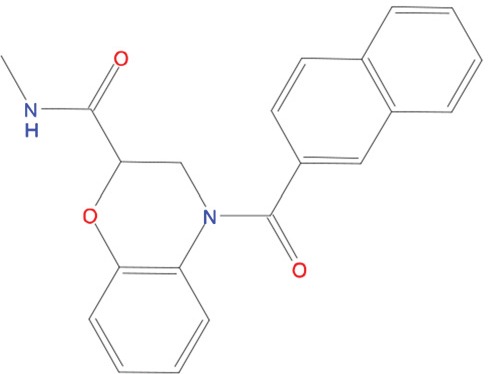	>100 μM
**C11**	14881755	349	-5.5	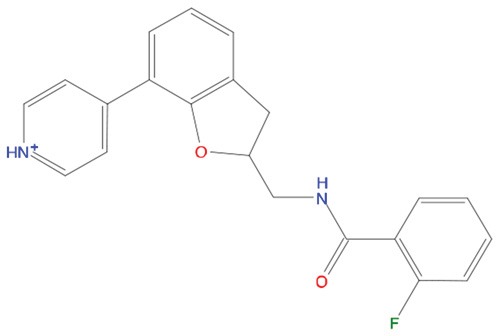	>100 μM
**C12**	09036647	422	-7.5	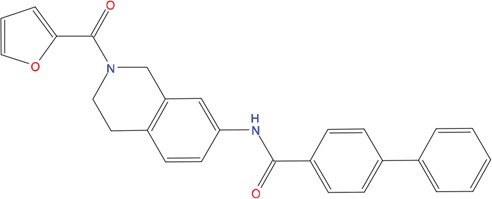	>100 μM
**C13**	10651845	423	-6.2	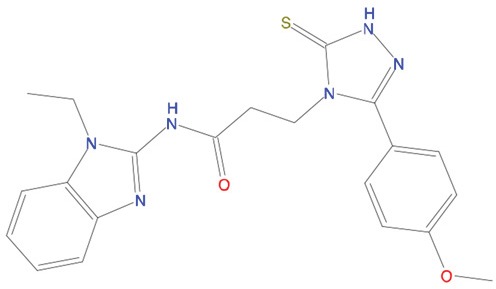	>100 μM
**C14**	10509523	441	-7.4	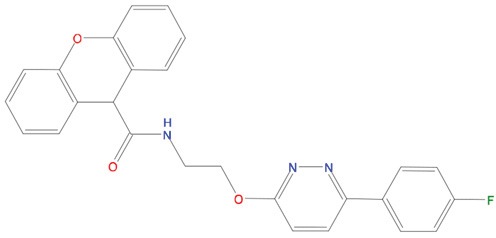	>100 μM
**C15**	10013689	453	-5.3	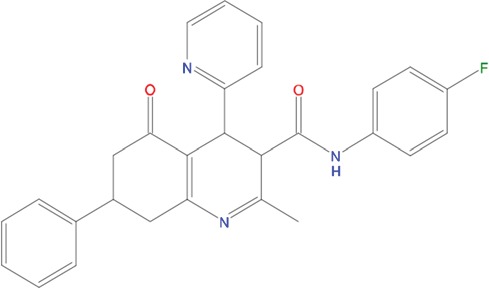	>100 μM
**C16**	08415310	454	-5.5	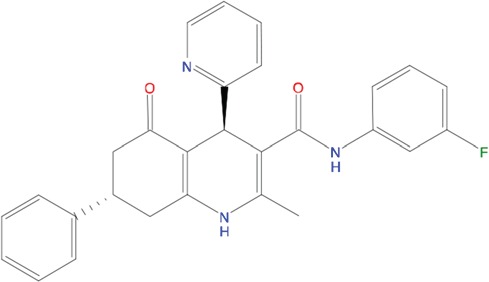	>100 μM
**C17**	12416384	460	-5.2	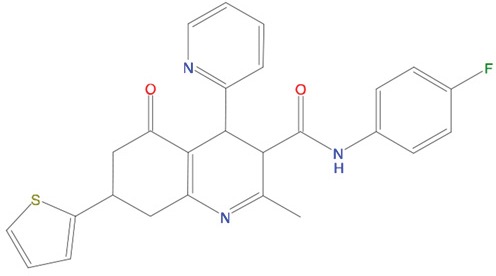	>100 μM
**C18**	12416388	460	-5.2	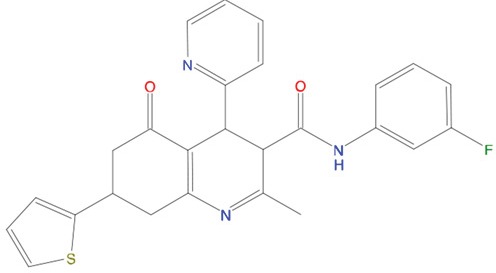	>100 μM
**C19**	09763292	461	-7.3	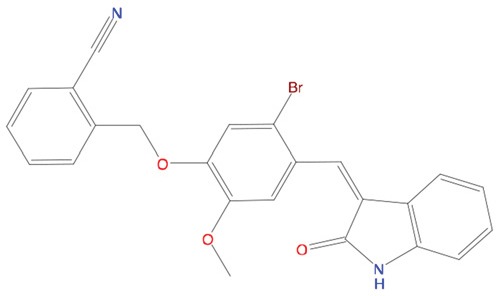	>100 μM
**C20**	09601607	464	-8.7	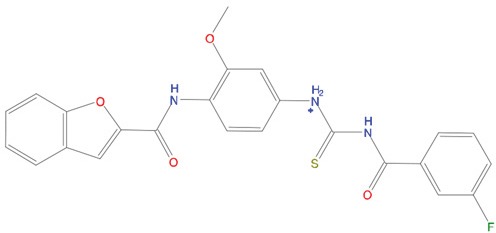	30±15 μM
**C21**	12522153	464	-7.3	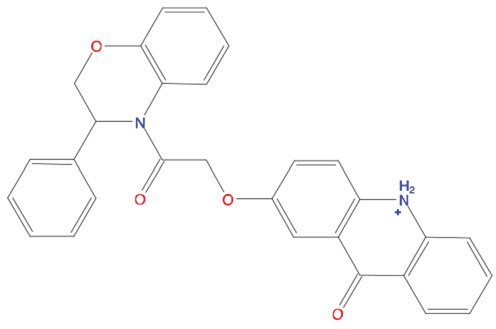	>100 μM
**C22**	08415461	470	-5.8	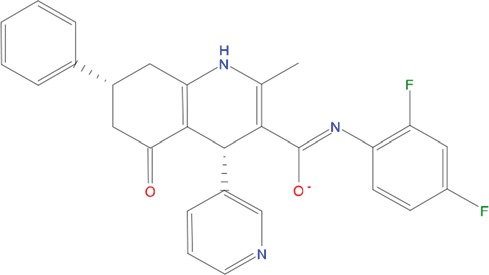	15±6 μM
**C23**	08792268	477	-7.4	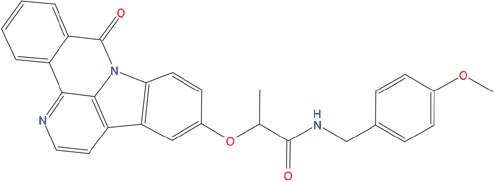	12±5 μM
**C24**	10578760	480	-6.8	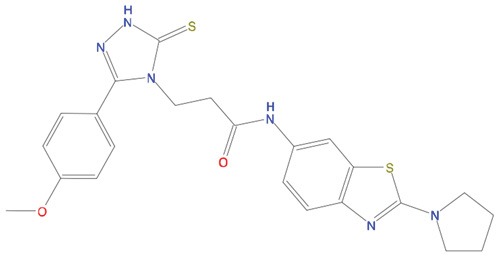	>100 μM
**C25**	08792436	491	-7.3	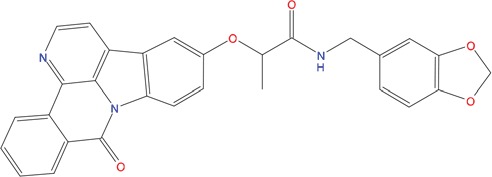	12±6 μM

When fitting of the sensorgrams for each compound yielded low affinity, the Kd are given as >100 μM. Standard errors of best-fit parameters are indicated.

### *In vitro* binding of selected compounds to recombinant Aurora-A

Solubility trials showed that most compounds have low solubility in aqueous conditions. However, all are soluble in 100% DMSO and remain soluble in running buffer (10 mM Hepes pH 7.4, 150 mM NaCl, 2% DMSO + 0.005% surfactant P20) at concentrations up to 100 μM.

**Figure 3:**
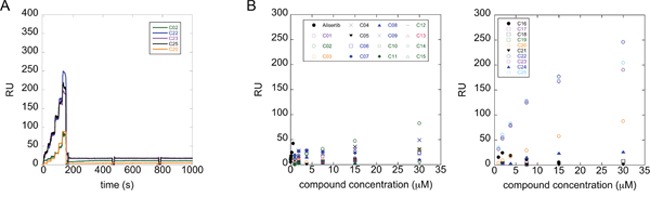
A subset of compounds directly binds Aurora-A *in vitro* Interaction of compounds with Aurora-A immobilized on a COOH5 sensorchip was measured by SPR experiments. Sensorgrams were obtained using Aurora-A as ligand and compounds (concentrations: 0-25 sec: 0.94 μM; 25-50 sec: 1.875 μM; 50-75 sec: 3.75 μM; 75-100 sec: 7.5 μM; 100-125 sec: 15 μM; 125-150 sec: 30 μM) as analytes. Panel A shows representative sensorgrams, for C02, C20, C22, C23 and C25; the increase in RU relative to baseline indicates complex formation upon injection of each compound, the decrease in RU represents dissociation of analytes from immobilized Aurora-A after injection of running buffer. Panel B shows the RU values upon injection of analytes at the indicated concentrations. Affinities, calculated from Scatchard plots from each compound, are reported in Table 1. Alisertib was used as positive control. Each sensorgram is the average of two experiments.

We evaluated the interaction of the selected compounds with Aurora-A immobilized on a COOH5 sensorchip by FastStep Surface Plasmon Resonance (SPR) experiments. Sensorgrams were obtained using Aurora-A as ligand and injecting increasing concentrations of the inhibitors (see Materials and Methods) as analytes in running buffer (Figure 3A). Formation of a complex between Aurora-A and compounds was indicated by the increase in resonance units (RUs) relative to baseline upon injection of each compound at each concentration; affinities were then calculated from Scatchard plots for each compound (Figure 3B), and are reported in Table 1. As a positive control, binding of the characterized Aurora-A inhibitor Alisertib was also followed (concentrations: 0-25 sec: 0.94 nM; 25-50 sec: 1.875 nM; 50-75 sec: 3.75 nM; 75-100 sec: 7.5 nM; 100-125 sec: 15 nM; 125-150 sec: 30 nM). As expected, Alisertib efficiently binds Aurora-A, with a resulting fitted Kd of about 40 nM. Interestingly, 5 out of the 25 tested compounds (C02, C20, C22, C23, C25) bound Aurora-A on chip with a Kd < 50 μM. Those were considered as the most promising hits and tested in further *in vitro* experiments.

### A subset of compounds is able to compete with TPX2 for binding to Aurora-A *in vitro*

**Figure 4:**
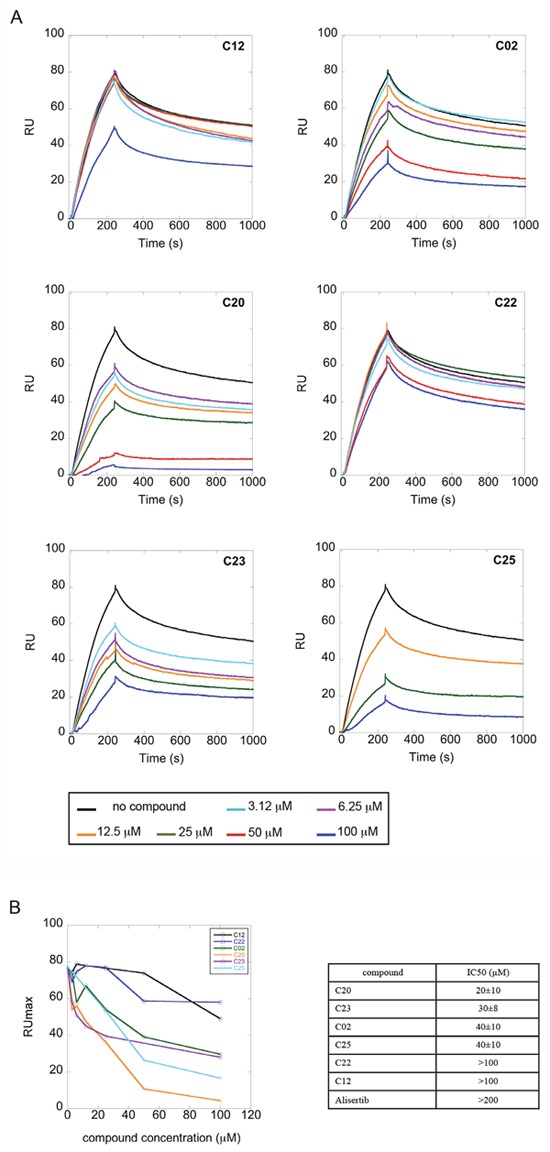
Competition experiments between GST-TPX2-1-43 and selected compounds for Aurora-A binding **(A)** Sensorgrams of competition experiments carried out on COOH5 chips with Aurora-A immobilized at a level of about 200 RUs, by injecting 1 μM GST-TPX2-1-43 together with increasing concentrations of compounds. **(B)** Fitting of competition experiments data. The decrease of TPX2 binding upon injection of increasing concentrations of competing compounds is shown. IC50 values of tested compounds are shown in the table on the right; standard errors of best-fit parameters are indicated.

**Figure 5:**
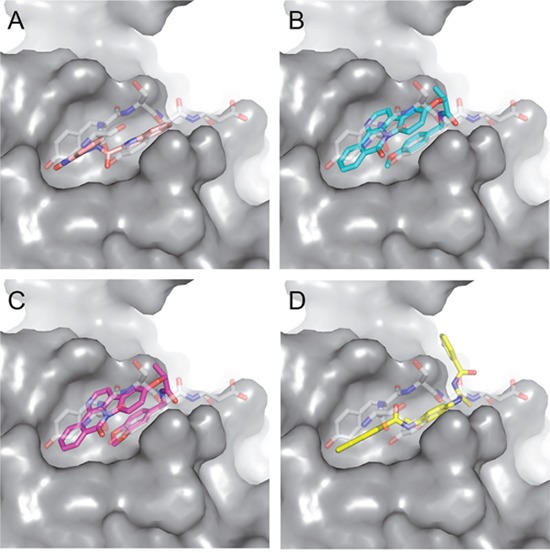
Predicted binding mode of compounds to Aurora-A The hydrophobic groove at the N-terminal lobe of Aurora-A is shown as grey surface. The position of TPX2-7-11 is shown in transparent sticks. Atom coloring is the following: N blue, O red, S orange. C02 **(A)**, C23 **(B)**, C25 **(C)** and C20 **(D)**.

We next asked whether the C02, C20, C22, C23 and C25 compounds that bound to Aurora-A with the highest affinity in SPR experiments could actually interfere with TPX2 binding to the kinase. Binding of a recombinant TPX2 fragment (amino acids 1-43, N-terminally tagged with GST) to Aurora-A was first evaluated to assess the experimental conditions for competition experiments. A Kd value of 80 nM was obtained, with very slow dissociation rate, indicating high complex stability (Supplementary Figure 1). Competition experiments were then carried out by simultaneously injecting increasing concentrations of the compounds and 1 μM GST-TPX2-1-43: sensorgrams are shown in Figure 4A, while the fitting of the experiments and the IC50 values of the analyzed compounds are reported in Figure 4B. Results indicate that 4 out of the 5 tested compounds decrease TPX2 binding to Aurora-A, thus behaving as competing compounds. The best inhibitors are C20 (IC50=20±10 μM), C23 (IC50=30±8 μM), C02 (IC50=40±10 μM) and C25 (IC50=40±10 μM), while C22 did not interfere with the formation of the Aurora-A/TPX2 complex. As a negative control, we tested in parallel the C12 compound that had shown no Aurora-A binding properties (Figure 3) and found that it did not influence the Aurora-A/TPX2 complex formation (Figure 4). Interestingly, Alisertib, which binds Aurora-A at the ATP-binding pocket, though binding to Aurora-A with high affinity (Figure 3) does not compete with TPX2 binding (Supplementary Figure 2 and Figure 4B). This observation corroborates the initial hypothesis based on docking analyses (Figure 5) that the newly selected compounds bind to the TPX2 binding pocket of the kinase.

### Effects of C20 and C23 treatment in osteosarcoma cells

**Figure 6:**
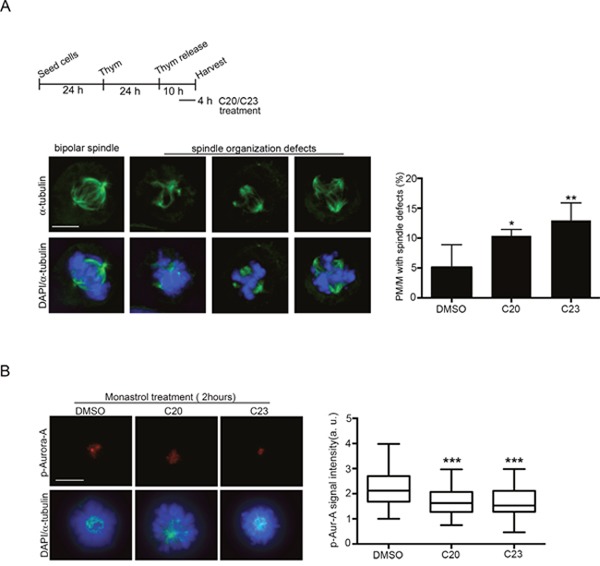
Effects of treatment with the C20 and C23 inhibitors on mitotic spindle structure and Aurora-A activity in U2OS cells **(A)** Top: schematic representation of the experimental protocol. The immunofluorescence panels (below) show a control spindle (left panel) and representative images of the observed defects in C20- or C23-treated cultures. Quantification is shown in the histograms on the right. About 300 prometa-metaphase (PM/M) cells per condition from 4-7 experiments were analyzed. **(B)** Representative immunofluorescence images of active p-Thr288-Aurora-A in control (DMSO) and C20- or C23-treated prometaphases. The distribution of signal intensity is quantified in the box plots on the right (n, at least 40 measured cells per condition, from 2 experiments). Fluorescence intensity is shown in arbitrary units (a.u.). *: p<0.05; **: p<0.01; *** p< 0.001; Student's t-test. Scale bars: 10 μm.

We next wished to examine how compounds displaying inhibitory activity on the Aurora-A/TPX2 complex formation *in vitro* affected mitosis in human cells. We decided to focus on the C20 and C23 compounds, which demonstrated the highest potency (see Figure 4B). C20 and C23 were tested in the U2OS osteosarcoma cell line, in which we previously characterized significant mitotic defects after Alisertib-dependent Aurora-A inhibition [7]. Briefly, U20S cells were arrested at the G1/S transition by thymidine treatment, released in fresh medium to progress through S and G2 phases, then treated with either C20 or C23 (10 μM) 6 hours after thymidine release (Figure 6A). After further 4 hours, samples were fixed and stained with antibodies against alpha-tubulin to visualize mitotic spindles assembled in the presence of the new Aurora-A/TPX2 inhibitors. Indeed, treatment with C20, and, to an even higher extent, with C23 yielded the appearance of aberrant mitotic spindles. Defects were scored in prometa- and metaphases and included completely disorganized spindles (Figure 6A, second panel), or spindles with supernumerary poles (Figure 6A, third and fourth panel), as typically observed under impaired Aurora-A function [7]. To directly assess the extent of Aurora-A activation we measured Aurora-A auto-phosphorylation on Thr 288. Cultures were synchronized by thymidine arrest/release as above, except that the Eg5 inhibitor monastrol was added 2 hours before fixation in order to increase the stringency of prometaphase arrest and compare cells at the very same stage of mitosis. Under these conditions, we measured a highly significant reduction in the p-Thr288-Aurora-A signal in cultures treated with both C20 and C23 compounds compared to controls (Figure 6B), confirming that both compounds impaired Aurora-A activation.

## DISCUSSION

Protein-protein interactions, an amazing reservoir of therapeutic targets, were once considered “undruggable”, because of their large and dynamic interfaces that make small molecule inhibitor design challenging [25, 26]. However, recent successful examples have questioned this view, and inhibitors of specific interactions have now reached clinical trials for anti-cancer therapy [27]. The frequent co-overexpression of Aurora-A and TPX2 in tumors suggests that they act as a whole tumorigenic unit [17], and direct evidence in aggressive tumors, supporting this hypothesis, is accumulating [28–30]. Therefore, we have undertaken a virtual screening approach in order to identify novel allosteric inhibitors of Aurora-A, acting via the disruption of the interaction with TPX2. Because the latter modulates Aurora-A at several levels (e.g., kinase activity, protein stability and localization at spindle MTs), the loss of TPX2 binding is expected to result in both functional inhibition and lowered levels of Aurora-A. The identification of PPI inhibitors of the Aurora-A/TPX2 complex may thus represent a promising strategy to open a novel therapeutic window for a specific subset of aggressive tumors.

Based on the solved structure of the Aurora-A/TPX2 complex [13], we have developed a rational approach to search for molecules that might disrupt the complex. This led to the identification of a hot-spot for drug design purposes comprising a shallow Aurora-A pocket formed by residues 160-200 as target site, and the 7-11 fragment of TPX2 as anchor site. Noteworthy, the residues of the identified anchor site were already pinpointed as key structural elements for TPX2 binding [31, 32]. Moreover, the same cleft was previously identified in other kinases strictly related to Aurora-A as a fundamental regulatory region, and termed the “PIF-pocket” [33]. The characterization of the PIF-pocket as a key regulatory site for enzyme activity paved the way to the development of compounds that allosterically modulate the catalysis of target kinases [33, 34]. Interestingly, by comparing one of the best structurally characterized allosteric inhibitors ((*5S,6S*)-7) of a homologous kinase to Aurora-A, i.e., human Pdk1 [34], to our hits, we noticed a strikingly similar feature, i.e., the presence of two aromatic rings anchoring to the PIF pocket (Supplementary Figure 3). In agreement with these studies, the ability to mimic the aromatic rings of Tyr 8 and Tyr 10 would therefore constitute an essential feature for an inhibitor of the Aurora-A/TPX2 complex formation. Indeed, the most potent compounds that we have identified share such a feature (Figure 5). Another interesting feature that emerged when a comparison between C20-C23 and compound (*5S,6S*)-7 was made (Supplementary Figure 3), is the “horseshoe” conformation of the ligand, which is necessary to bury the aromatic rings of the inhibitor deep inside the PIF pocket of the kinase.

SPR data indicated that five out of the 25 tested compounds are able to bind Aurora-A at medium/low micromolar values. Most importantly, four of them also interfere with the binding of TPX2 to Aurora-A *in vitro*, in contrast with the classical type-I inhibitor of Aurora-A, i.e., Alisertib, which does not affect TPX2 binding. Although our assays do not yet pinpoint the actual binding site of compounds, they strongly suggest that there is a direct competition between TPX2 and the identified hits for binding the PIF pocket of the kinase.

The two most promising molecules were administered to human cells that were synchronously progressing towards mitosis: both induced a significant fraction of prometa- and metaphases to assemble mitotic spindles with severely altered structure, even at a concentration of 10 μM. Specifically, the induction of supernumerary spindle poles or disorganized spindles, highly reminiscent of those generated under Aurora-A inhibition [7, 35–36], suggests that the identified molecules are targeting Aurora-A function in cultured tumor cells. Reduced Aurora-A kinase activity was indeed measured, by using auto-phosphorylation on Thr 288 as a read out.

While this manuscript was in preparation, results from a high-throughput screening of 2×10^5^ compounds, aimed at identifying Aurora-A/TPX2 interaction inhibitors, were published [37]. Interestingly, the AurkinA molecule identified in [37], although chemically diverse from our hits, binds the kinase at the PIF pocket (the so called “Y-pocket”), supporting the soundness of our premises. The insights coming from our study and [37] could be merged and exploited in future drug design studies to refine our initial PH and identify new, more potent compounds. The present study offers two additional valuable indications: first, it corroborates the emerging notion that virtual screening approaches can hugely decrease the number of compounds to be tested *in vitro* for a potentially successful drug discovery campaign. Second, it indicates that molecules solely targeting the Aurora-A/TPX2 interaction surface are sufficient to effectively disrupt mitotic processes that are dependent on Aurora-A activity.

It must be noted that in both studies, cellular assays were carried out at quite high inhibitor concentrations (10 μM in this study, >50 μM in [37]). We can speculate that this was necessary because of the high affinity between Aurora-A and TPX2 (Kd value in the nanomolar range in our *in vitro* assays) and the stability of the complex. The low solubility in aqueous solution of the compounds tested in this study is an additional issue limiting their usefulness for drug discovery purposes. Further optimization will be required for the design of a new generation of Aurora-A inhibitors. This will involve the improvement of the solubility and cell permeability by rational-based modifications of the original scaffold (e.g., carrier-linked prodrugs and bioprecursors [38]), and non-amide backbone replacements in C20 and C23 to improve their stability and resistance to potential proteolysis. Inclusion complex formation-based techniques [39] are also currently under investigation, using cyclodextrins as host molecules.

Besides providing interesting scaffolds for the development of anti-cancer drugs, overall the results represent a proof-of-concept that kinases can be targeted by disrupting their interaction with specific activators. Thus, novel types of kinase inhibitors with distinct features in terms of specificity of action and chemical diversity may be designed, of potential interest in the therapeutic field.

## MATERIALS AND METHODS

### Identification of hot spots at the Aurora-A/TPX2 interaction interface

The crystal structure of the kinase domain (residues 122-403) of human Aurora-A bound to the 1-43 TPX2 fragment [13] was used as query in Consurf [18], CAMPO [19] and computational Alanine Scanning Mutagenesis (ASM) [20] servers. CAMPO and Consurf are web servers for the automatic identification and mapping of the level of evolutionary conservation of residues of a query protein. Starting from the latter, the servers automatically collect homologues, infer their multiple sequence alignment and, within a probabilistic framework, estimate the evolutionary rates of each sequence position. For both servers, adjustable parameters for homologous sequences retrieval and evolutionary score assignment were kept at their default values. The obtained values of evolutionary conservation were then normalized with a conservation score scale (0, highly variable; 9, invariant) and the mean of the values obtained from the two servers were computed. The computational ASM server measures the change in binding free energy of Gibbs (ΔΔG) for the replacement of an amino acid side chain with Alanine. Positive values mean that replacement by Alanine is predicted to destabilize the complex; negative values predict a stabilizing effect.

### Ligands library and target preparation

The drug-like (~5×10^6^ compounds) and lead-like (~3×10^6^ compounds) subsets of the ZINC database [21] were downloaded to generate low-energy conformations. The “Conformation import” function of MOE (The Molecular Operating Environment; Chemical Computing Group®, Montreal, Canada) was applied to this end. This function performs detailed conformational search either on the currently loaded molecular system or a given molecular database. For each input molecular system, the following algorithm is used to generate conformations: 1) perturb the coordinates of the atoms with Systematic Search (SS); SS will systematically rotate all non-ring bonds in fixed increments; 2) energy minimize conformers of step 1) with the MMFF94x Forcefield for a maximum of 200 iterations, or until the specified root mean square gradient of 0.001 has been reached; 3) retain only the conformers with strain energy less than 4 kcal/mol. An RMSD criterion is used for duplicate detection (RMSD < 0.15 Å); optimal rotation and translation are applied for systems with no fixed atoms. For each compound, the following settings were also applied: maximum 250 conformers for each compound; no input filters; constraints options kept at their default values. The obtained conformations were saved as a Moe Database Format (.mdb file).

The three-dimensional (3D) structure of Aurora-A in complex with TPX2 was downloaded from PDB (accession number 1OL5). All other ligands except TPX2 were removed by manually editing the PDB file. The protonation state and geometry of residues of 1OL5 were assigned using the “Protonate 3D” function of MOE. Protonate 3D solves the macromolecular protonation state assignment problem by selecting a protonation state for each chemical group that minimizes the total free energy of the system (taking titration into account). Finally, the complex was visually inspected in order to verify the absence of steric clashes between TPX2 and the residues at the binding site.

### Pharmacophore model generation and search

Pharmacophore modeling calculations were performed using MOE. The MOE “Pharmacophore Query” (PQ) was used in order to build the initial Pharmacophore Hypothesis (PH), starting from the complex between Aurora-A and the identified hot spot of interaction of TPX2 (TPX2-7-11). Through the “Unified Annotation Scheme”, the pharmacophore annotation points (such as H-bond donor, H-bond acceptor, hydrophobic) were assigned. MOE's default values for the radii of atom-based chemical features (1.0 Å) and projections (1.4 Å) were used. An excluded volume, comprising residues at the active site of Aurora-A located within a radius of 4 Å from any atom of TPX2-7-11, was also included in the final PH. The latter was used to screen the ZINC libraries of compounds previously obtained, using the “Pharmacophore Search” option of MOE. All features of Tyr 8 and Tyr 10 were set as essential; compounds not satisfying at least three features of the PH were discarded.

### Docking of compounds

Compounds retrieved from the pharmacophore search were docked into the binding cleft of Aurora-A, by means of DOVIS 2.0 [22]. The 3D structure of Aurora-A was prepared by automatically assigning bond orders and hybridization, charges, and Tripos atom types. The previously obtained explicit hydrogens during target preparation were kept. An energy grid with 40×40×40 (numbers refer to the number of grid points in xyz), centered on the TPX2-7-11 binding cleft of Aurora-A, and a 0.375-point spacing were used. Atom-specific affinity maps were then computed for each atom observed in the ligands, and a distance-dependent dielectric constant was adopted. Several docking simulations were initially run. Before screening the compounds, grid dimension and center values were systematically varied (other parameters were maintained at their default values) in order to find which combination of values was able to best reproduce the binding mode of TPX2-7-11 to Aurora-A in the crystal structure. The selected values for grid dimensions and center were respectively 30×15×15 and x=185.0, y=190.0, z=11.0 (coordinates are referred to the 1OL5). During the high-throughput docking, the Lamarckian Genetic Algorithm was chosen as search algorithm, using the following values: number of individuals in population, 150; maximum number of energy evaluation, 250000; maximum number of generation, 27×10^3^; rate of gene mutation, 0.02. Water molecules were excluded from the docking calculations. All other parameters were kept at their default values. Each compound was ranked according to its score, obtained with the scoring function implemented in DOVIS [22], which is mainly based on steric and electrostatic interactions with the target site. The pose with the highest score for each compound was selected for post-filtering. To perform the above mentioned dockings, approximately 35000 h/CPU were used, using the HPC clusters provided by CINECA. During the post-filtering phase, the mean and standard deviation of the docking scores were then calculated, and those compounds scoring at >2.0 standard deviation from mean (1032 Drug-like and 2413 Lead-like compounds) were selected and re-docked with Autodock Vina [23] and MolDock SE [24]. When Autodock Vina was used, the same grid parameters described previously were adapted, the exhaustiveness option was set to 8, and all other options were kept at their default values. When MolDock SE was used, a search space with a 15 Å radius, centered on the TPX2-7-11 binding cavity and the grid-based MolDock score with a grid resolution of 0.30 Å were used. Only those compounds showing a similar (RMSD<2.0 Å, not considering hydrogens) docked pose, as assessed by Dovis, AutoDock Vina and MolDock, were kept. *Ad-hoc* Python scripts were used to measure the RMSD (available from authors upon request). Finally, the selected poses were filtered with the previously developed PH with the “absolute position” option of MOE activated. In this way, when analyzed during PS, the poses were not allowed to be translated or rotated, and only those in agreement with the PH were kept.

### Recombinant protein expression and purification

Recombinant His-tagged Aurora-A was either of commercial source (Life Technologies) or expressed and purified from bacterial cultures. Recombinant GST protein alone was from Sigma (SRP5348). Plasmids for the expression of Aurora-A or the 1-43 fragment of TPX2, subcloned in frame with N-terminal His-tag or GST, respectively, (kind gift from Richard Bayliss), University of Leeds, UK were transformed into BL21(DE3) *Escherichia coli* strain.

An overnight growth of 15 ml was diluted 1:100 and grown at 37°C in Luria Bertani (LB) medium containing 30 μg/ml kanamicin for 3 hours (O.D._600_≈0.6); expression of the protein was then induced with 0.2 mM IPTG (isopropyl β-d-thiogalactoside) for 20 hours at 20°C, 200 rpm. Cells were collected after centrifugation (10 min at 12000 rpm), resuspended in 40 ml of buffer A (50 mM Hepes pH 7.5, 300 mM NaCl, 5 mM MgCl_2_, 10 % glycerol) in the presence of Protease Inhibitor Cocktail cOmplete® (Roche) and lysed by sonication.

After centrifugation, Aurora-A was found mainly in the supernatant and purified using a Ni-HiTrap™ Chelating HP column (GE-Healthcare) equilibrated with buffer A; the protein was eluted with buffer A plus 300 mM imidazole, after column wash at 100 mM imidazole. Fractions containing purified Aurora-A were pooled and further purified by SEC (Superdex 75 16/30, in buffer A), to remove aggregates; the final yield was 5 mg/l of culture of Aurora-A purified to homogeneity. The extinction coefficient at 280 nm was determined by the BCA assay (Sigma) to be 34 mM^−1^cm^−1^ (per monomer).

For the 1-43 fragment of TPX2, the supernatant fraction was loaded onto a GSTrap™ HP column (GE-Healthcare) equilibrated with PBS buffer at pH 7.3; after extensive washing with PBS, the protein was eluted with 50 mM Tris HCl pH 8.0 plus 30 mM glutathione (Flow: 1 ml/min), and extensively dialyzed *vs* PBS buffer at pH 7.3. The final yield was 2 mg/l of culture of purified TPX2 fragment.

### Surface plasmon resonance (SPR) experiments

SPR experiments were carried out using a SensiQ Pioneer apparatus. The sensor chip (COOH5, with a hydrogel-based sensing surface endowed with high binding capacity) was chemically activated by a 35 μl injection of a 1:1 mixture of 200 mM N-ethyl-N’-(3-diethylaminopropyl) carbodiimide and 50 mM N-hydroxysuccinimide at a flow rate of 5 μl/min. Aurora-A was immobilized on the activated sensor chip via amine coupling. The immobilization was carried out in 20 mM sodium acetate at pH 4.5; the remaining ester groups were blocked by injecting 1 M ethanolamine hydrochloride (35 μl). This procedure ensures immobilization of Aurora-A principally via the N-terminus.

Compounds for testing were purchased from Ambinter (Paris, France). All chemicals were of the highest purity available, as certified by vendor. For the experiment of direct binding of Aurora-A to compounds, about 18000 resonance units (RUs) of Aurora-A were immobilized onto both Fc1 and Fc2 flow cells, leaving the empty Fc3 flow cell as control. Compounds were injected onto the sensor chip at a constant flow in FastStep injection experiments, in order to rapidly obtain information on the binding of the analyte at different concentrations, with a single dissociation phase. Compounds were diluted in 10 mM Hepes pH 7.4, 150 mM NaCl, 2% DMSO + 0.005% surfactant P20 (running buffer) and injected by 6 serial doubling steps (nominal flow rate = 200 μl/min) onto flow cells, at the following concentrations: 0-25 sec: 0.94 μM; 25-50 sec: 1.875 μM; 50-75 sec: 3.75 μM; 75-100 sec: 7.5 μM; 100-125 sec: 15 μM; 125-150 sec: 30 μM or by 7 serial doubling steps, at the following concentrations: 0-25 sec: 0.94 μM; 25-50 sec: 1.875 μM; 50-75 sec: 3.75 μM; 75-100 sec: 7.5 μM; 100-125 sec: 15 μM; 125-150 sec: 30 μM; 150-162 sec: 60 μM. At least two independent experiments were performed and averaged for each series of injections. The interaction of immobilized Aurora-A ligand with the inhibitor compounds was detected through mass concentration-dependent changes in the refractive index on the sensor chip surface, expressed as RUs.

Binding of GST-TPX2-1-43 to His-Aurora-A was assayed using about 200 RUs of Aurora-A immobilized onto both Fc1 and Fc2 flow cells, leaving the empty Fc3 flow cell as control. TPX2 in running buffer was injected at different concentrations (ranging from 10 nM to 5 μM), in order to determine the affinity constant of the interaction between Aurora-A and TPX2 and the conditions of the competition experiments (flow rate= 30 μl/min). For the latter, different concentrations of the compounds which were demonstrated to interact with Aurora-A with the highest affinity were injected together with 1 μM GST-TPX2-1-43 on the sensor chip at a constant flow (flow rate= 30 μl/min) in running buffer, taking advantage of the different molecular mass of compounds *vs* TPX2, which is about 50-100 higher than those of the inhibitor compounds. IC50 were calculated on the basis of the RU value at each inhibitor concentration (10 sec after the end of each association sensorgram) since TPX2 binding signal decreases by increasing inhibitor concentration. Both rapid binding and rapid dissociation of inhibitors were subtracted from the sensorgrams.

When indicated, the Alisertib (Selleck Chemicals) Aurora-A inhibitor was used as reference.

### Cell cultures, synchronization protocols and treatments

The human U2OS osteosarcoma cell line (ATCC: HTB-96) was grown at 37°C in a 5% CO_2_ atmosphere in DMEM, supplemented with 10% fetal bovine serum. For synchronization, cells were subjected to a 24 hours block in 2 mM thymidine. Cultures were then released from the G1/S arrest by washing away the thymidine and adding fresh medium containing 30 μM deoxycytidine; 6 hours post-release cultures were treated with the C20 or C23 compounds at the indicated concentration. Mock-treated cultures were incubated with DMSO. After further 4 hours cells were harvested and analyzed. When indicated, monastrol (100 μM, TOCRIS, cat. 1305) was added 2 hours before fixation.

### Immunofluorescence (IF) microscopy

Cells grown on poly-lysine-coated coverslips were fixed with cold methanol, 6 min at -20°C. Blocking and all antibody incubations were performed at room temperature in PBS/0.05% Tween 20/3% BSA. Cells were counterstained with 4,6-diamidino-2-phenylindole (DAPI, 0.1 μg/ml) and mounted using Vectashield (Vector Laboratories). Primary antibodies were: chicken anti-alpha-tubulin (1:50, Abcam), mouse anti-alpha-tubulin (1:2000, B-5-1-2, Sigma Aldrich), rabbit anti p-Thr288-Aurora-A (1:100, C39D8; Cell Signaling Technology). FITC- or Cy3-conjugated secondary antibodies were from Jackson ImmunoResearch Laboratories. Samples were analyzed using a Nikon Eclipse 90i microscope equipped with a Qicam Fast 1394 CCD camera (QImaging). Images were acquired using a 40x objective (PlanFuor, N.A. 0.75, Nikon) or a 100x objective (PlanFluor, Oil Immersion, N.A. 1.3, Nikon) and the Nis-Elements AR 3.2 software. Deconvolution, Extended Depth of Focus and Maximum Intensity Projections from Z-serial optical sections (range: 8 μm; 0.8 μm stacks when using the 40x objective; 0.4 μm stacks when using the 100x objective) were performed using Nis-Elements HC 4.2 (Nikon); images were further processed with Adobe Photoshop CS 8.0. The intensity of the p-Thr288-Aurora-A signal was measured using Nis Elements HC 4.2 on Maximum Intensity Projections from acquired z-stacks. Sum intensity values measured in the whole cell were normalized within each experiment putting as “1” the lower value of control cells; data were statistically analyzed using Graph Pad Prism 6.

## SUPPLEMENTARY MATERIALS FIGURES


