# Supplementary material for: Identification of small molecule inhibitors of the Aurora-A/TPX2 complex
Source: Oncotarget. 2017 Mar 31;8(19):32117–33. doi: 10.18632/oncotarget.16738 (PMC5458272; doi:10.18632/oncotarget.16738)
Supplement: Supplementary file 1 [file oncotarget-08-32117-s001.pdf]

Identification of small molecule inhibitors of the Aurora-A/TPX2 complex

SUPPLEMENTARY FIGURES

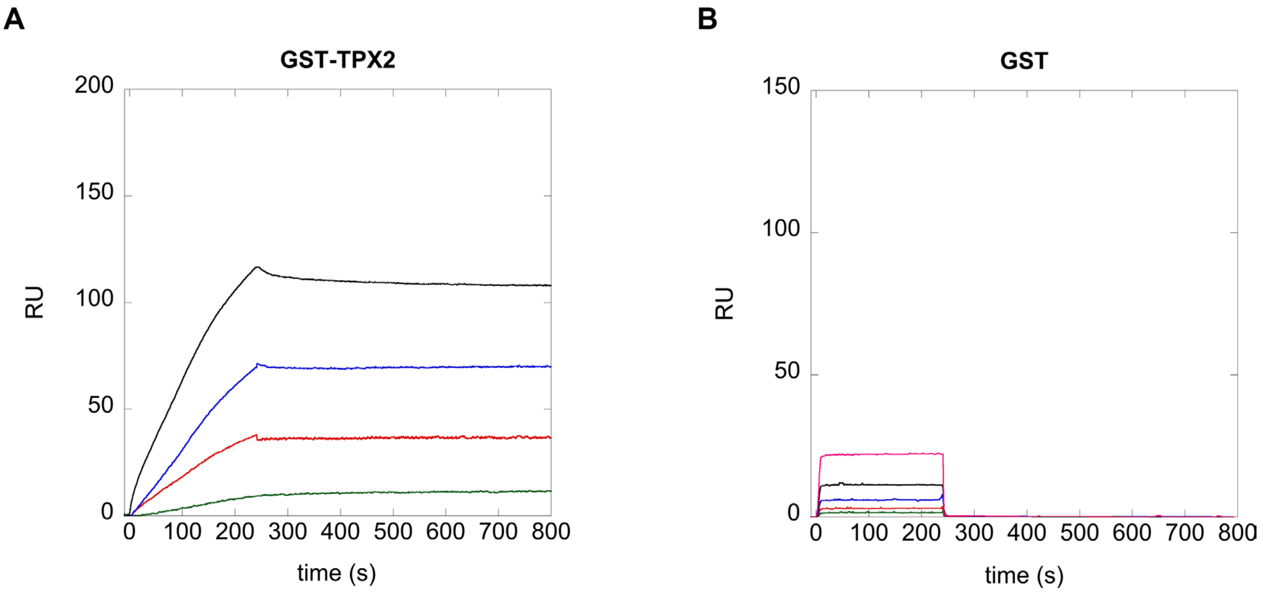

Supplementary Figure 1: Aurora-A interacts with high affinity and specificity with TPX2 (1-43). SPR sensorgrams showing the interaction of Aurora-A, immobilized onto a COOH5 sensorchip, with GST-TPX2-1-43 **(A)** or GST alone as a control **(B)** analytes at different concentrations. The increase in RU relative to baseline indicates complex formation between the immobilized Aurora-A ligand and the analytes. The plateau region represents the steady-state phase of the interaction. The decrease in RU after 240 sec indicates analyte dissociation from the immobilized Aurora-A upon buffer injection. Analyte concentrations: 0.3 µM (green), 0.6 µM (red), 1.2 µM (blue), 2.4 µM (black), 4.8 µM (magenta). GST-TPX2-1-43: Kd = 80 nM; GST: Kd > 5 µM.

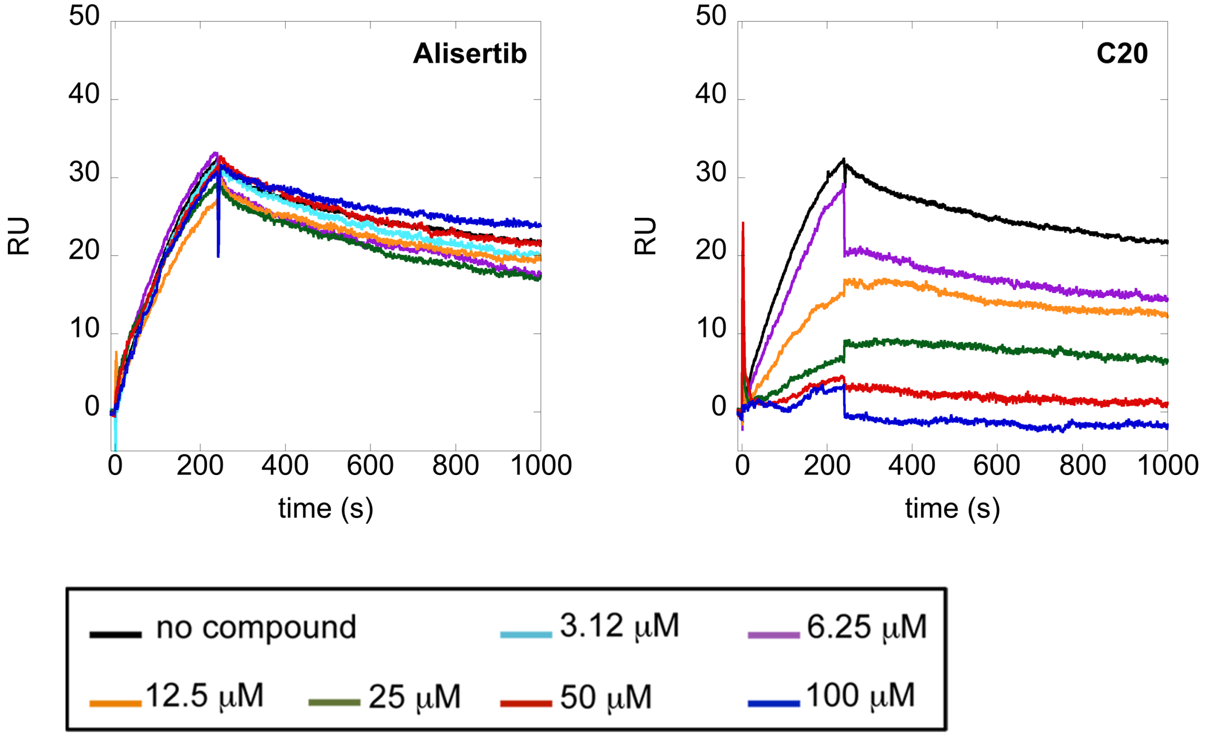

Supplementary Figure 2: Alisertib does not interfere with TPX2 binding to Aurora-A. Sensorgrams of SPR competition experiments carried out on COOH5 chips with Aurora-A immobilized at a level of about 200 RUs, by injecting 500 nM GST-TPX2-1-43 together with increasing concentrations of either Alisertib or the C20 compound.

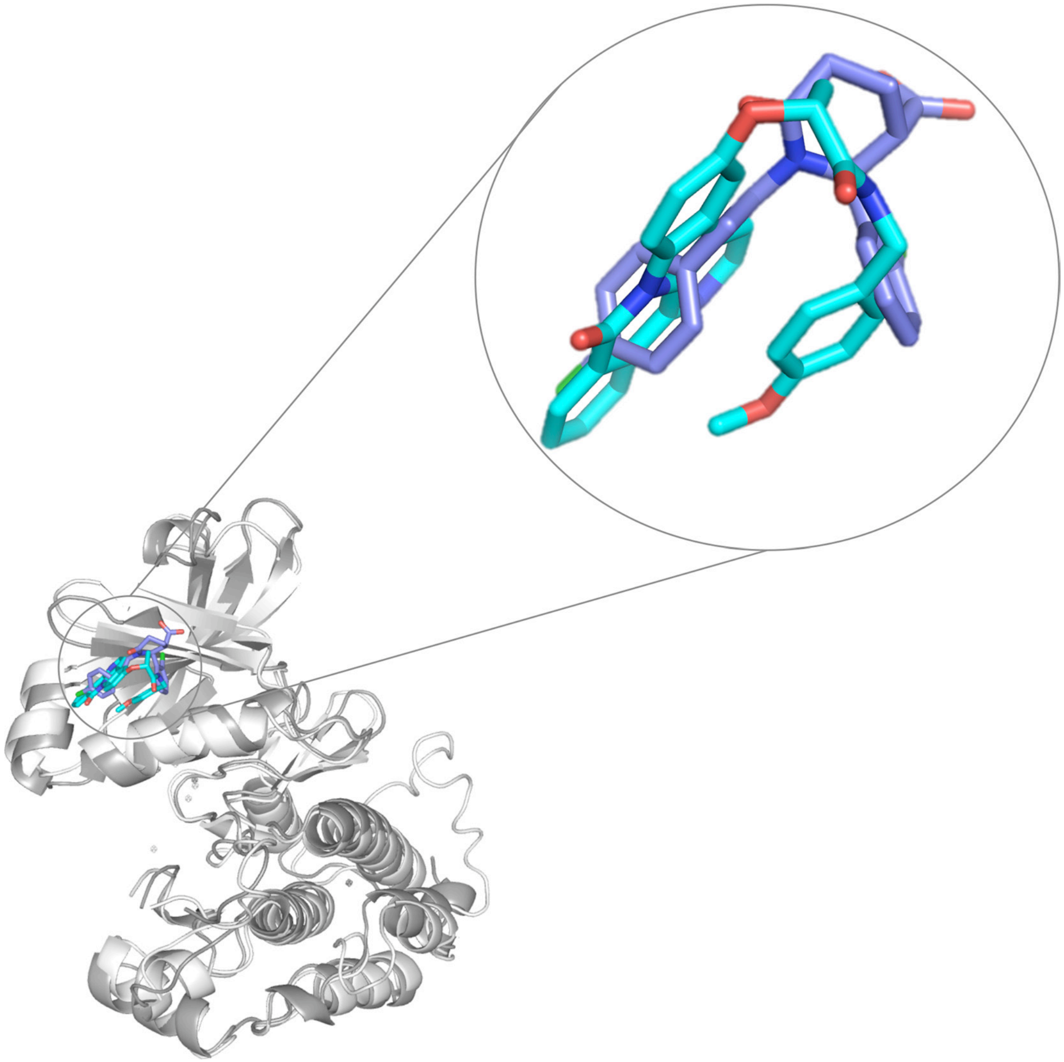

Supplementary Figure 3: Comparison between the proposed binding mode of C23 (cyan) to Aurora-A (white cartoons) and the crystal structure of the allosteric inhibitor 7 (slate) to Human Pdk1 Kinase (grey cartoons; PDB: 5ACK).
